# Analysis of Rejection, Infection and Surgical Outcomes in Type I Versus Type II Diabetic Recipients After Simultaneous Pancreas-Kidney Transplantation

**DOI:** 10.3389/ti.2024.13087

**Published:** 2024-09-19

**Authors:** Eric J. Martinez, Phuoc H. Pham, Jesse F. Wang, Lily N. Stalter, Bridget M. Welch, Glen Leverson, Nicholas Marka, Talal Al-Qaoud, Didier Mandelbrot, Sandesh Parajuli, Hans W. Sollinger, Dixon B. Kaufman, Robert R. Redfield, Jon Scott Odorico

**Affiliations:** ^1^ Anette C and Harold C Simmons Transplant Institute, Baylor University Medical Center, Dallas, TX, United States; ^2^ Division of Transplantation, Department of Surgery, School of Medicine and Public Health, University of Wisconsin-Madison, Madison, WI, United States; ^3^ Department of Medicine, School of Medicine, Creighton University, Omaha, NE, United States; ^4^ Department of Surgery, School of Medicine and Public Health, University of Wisconsin-Madison, Madison, WI, United States; ^5^ Division of Nephrology, Department of Medicine, School of Medicine and Public Health, University of Wisconsin-Madison, Madison, WI, United States

**Keywords:** infection, rejection, complication, pancreas-kidney transplantation, type 2 diabetes

## Abstract

Given the increasing frequency of simultaneous pancreas-kidney transplants performed in recipients with Type II diabetes and CKD, we sought to evaluate possible differences in the rates of allograft rejection, infection, and surgical complications in 298 Type I (T1D) versus 47 Type II (T2D) diabetic recipients of simultaneous pancreas-kidney transplants between 2006-2017. There were no significant differences in patient or graft survival. The risk of biopsy-proven rejection of both grafts was not significantly different between T2D and T1D recipients (HR_pancreas_ = 1.04, p = 0.93; HR_kidney_ = 0.96; p = 0.93). Rejection-free survival in both grafts were also not different between the two diabetes types (p_pancreas_ = 0.57; p_kidney_ = 0.41). T2D had a significantly lower incidence of *de novo* DSA at 1 year (21% vs. 39%, p = 0.02). There was no difference in T2D vs. T1D recipients regarding readmissions (HR = 0.77, p = 0.25), infections (HR = 0.77, p = 0.18), major surgical complications (HR = 0.89, p = 0.79) and thrombosis (HR = 0.92, p = 0.90). In conclusion, rejection, infections, and surgical complications after simultaneous pancreas-kidney transplant are not statistically significantly different in T2D compared to T1D recipients.

## Graphical Abstract

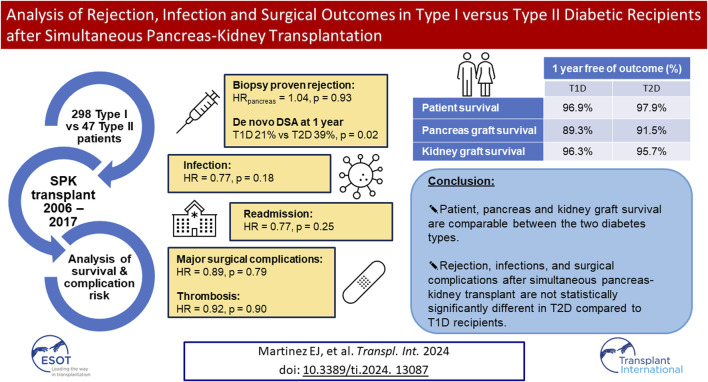

## Introduction

Simultaneous pancreas-kidney transplantation (SPKT) in Type I diabetes (T1D)with end-stage renal disease (ESRD) has produced significant improvement in prolongation and quality of life. Patient survival approaches 97% and 92% at 1 year and 3 years, respectively [1]. The half-life of pancreas allografts has increased to 15.5 years [2] secondary to advances in immunosuppressive therapy, surgical techniques, and immune monitoring [1, 3–5]. SPKT is also associated with improved kidney graft survival [6, 7] and improved preservation of kidney graft ultrastructure and function [8] compared to deceased donor kidney transplant alone.

Concerning SPKT in Type II diabetes mellitus (T2D) patients with ESRD, many studies have addressed the outcomes of pancreas transplantation for such patients [9]. Such studies have found comparable results between the two types of recipients regarding various endpoints including insulin resistance and β-cell function [3], kidney and pancreas graft survival [9–16], post-transplant glycemic control, BMI control [9, 17], and patient survival [6, 11, 18].

However, the effect of diabetes type on graft rejection after pancreas transplantation is less well understood. Differing rates of allograft rejection are observed in other abdominal solid organ transplants based on the primary etiology of the organ failure, especially with autoimmune components [19–30]. Several studies have evaluated the effects of donor-specific anti-HLA antibodies (DSA) on graft outcomes [31–33] and noted significantly decreased kidney and pancreas allograft survival [33–36]. None of these studies, however, account for the type of diabetes as a distinguishing factor.

In addition, T2D patients may be obese and consequently may have an increased risk of surgical site infections [37, 38] and worse graft outcomes [39]. The inflammatory milieu of T2D may impact the risk of surgical infections, thrombosis, etc., [40–42]. While these theoretical risks may exist, the outcomes of T1D and T2D SPKT recipients with respect to important specific surgical and infection-related outcomes have not been thoroughly evaluated.

Thus, in this study, we sought to comprehensively examine whether the type of diabetes impacts the rates of acute biopsy-proven rejection and DSA development as well as other key surgical and infectious complications. Additionally, we globally analyze factors contributing to these outcomes in the T1D and T2D SPKT populations.

## Material and Methods

**FIGURE 1:**
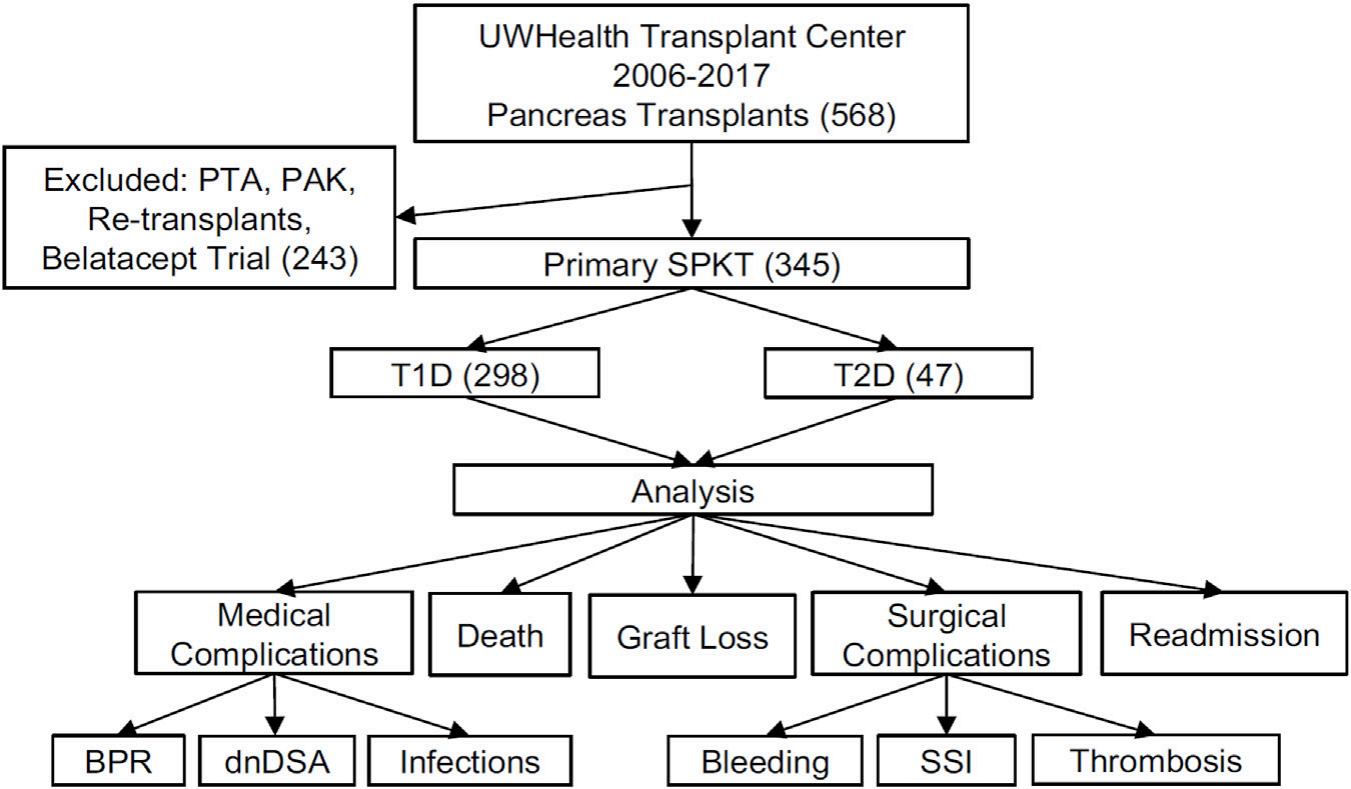
Schematic diagram of study design and data analysis. UWHC, University of Wisconsin Hospital and Clinics; PTA, Pancreas Transplant Alone; PAK, Pancreas After Kidney (transplant); SPKT, Simultaneous Pancreas Kidney Transplant; PTA, Pancreas Transplant Alone; PAK, Pancreas After Kidney (transplant); T1D, Type 1 Diabetes Mellitus; T2D, Type 2 Diabetes Mellitus; BPR, Biopsy Proven Rejection; dnDSA, *de novo* Donor Specific Antibody; SSI, Surgical Site Infection.

A single center retrospective review of prospectively collected data from a comprehensive in-house Transplant Database, electronic medical records, and the UNOS/OPTN STAR file was approved by the local Institutional Review Board. Analysis included primary SPKT recipients from 2006–2017 with 1-year minimum post-transplant follow-up. Diabetes mellitus types were determined by a holistic assessment with a grading system that included factors of patients’ age at diabetes onset, need for immediate use of insulin, pre-transplant fasting C-peptide, family history of diabetes, and the presence of autoantibodies (GAD65, Insulin- and Islet-antibodies) [43]. Primary outcomes included patient and graft survival, incidence of biopsy proven pancreas and kidney rejection and dnDSA, readmissions, infections, and surgical complications, including bleeding, pancreatic graft thromboses and other surgical site complications (Figure 1).

### Clinical Management

Systemic venous drainage and enteric exocrine drainage were performed in all SPKTs. Most patients were transferred to the transplant floor post-operatively with aspirin as the sole anticoagulation and without NG tube placement. Each patient’s immunosuppressive therapy was protocolized based on pre-transplant immunologic risk assessment. Either Alemtuzumab (ALEM) (30 mg, 1 dose), anti-thymocyte globulin (ATG) (1.5 mg/kg, 3-4 doses), or basiliximab (BAS) (20 mg, 2 doses) were used for induction therapy. Oral tacrolimus (initial target levels 8–10 ng/mL in the first year and 6–8 ng/mL thereafter) and oral mycophenolic acid (720 mg twice daily) were used as maintenance therapy in all patients. Dexamethasone 100 mg IV was administered intraoperatively and tapered to prednisone thereafter per protocol. Post-induction, selected patients underwent either early steroid withdrawal protocol or a rapid steroid taper to prednisone 5 mg daily by 1 month. All recipients receiving BAS induction received a more delayed steroid taper to prednisone 5–10 mg daily by 6 months. Nystatin and trimethoprim-sulfamethoxazole were given for 3 months and 1 year respectively. CMV prophylaxis with valganciclovir or acyclovir was given for 6 and 3 months depending on the recipients’ risk. Virtual crossmatching has been our standard minimal compatibility testing for the entire study period.

### Outcome Definitions

#### Graft Failure

Per UNOS definitions, pancreas graft failure was defined by graft pancreatectomy, reregistration for pancreas transplant, registration for islet transplantation, use of insulin >0.5 unit/kg/day for 90 consecutive days, or recipient death. Kidney graft failure was defined by graft nephrectomy, return to maintenance dialysis, or recipient death [44].

#### Graft Rejection

Pancreas allograf biopsy indications included post-transplant elevation of amylase or lipase, DSA increase or dnDSA, and hyperglycemia. Pancreas and kidney biopsies were evaluated by light microscopy with assignment of a grade (indeterminate/borderline, I, II, and III) and degree of immunohistochemical staining for C4D (none, <5%, or >5%) according to the Banff grading schema [45]. Acute rejection outcome represents cellular rejection or antibody mediated rejection or both.

#### De Novo DSA

Donor-specific anti-HLA Class I and II antibodies were detected pre- and post-transplant using Luminex single antigen beads (One Lambda, Canoga Park, CA). Antibodies were identified using multiple criteria including patterns of epitope reactivity, mean fluorescence intensity (MFI) value, specific bead behaviors, and assay background [46]. Since 2014, routine post-transplant monitoring of DSA has been performed on all transplant recipients at 6 and 12 months, and annually thereafter. Patients with a pretransplant calculated panel reactive antibody greater than zero were tested at an additional 6-week time point, and patients with pre-transplant DSA were tested at additional 3-week, 6-week, and 3-month time points. All patients undergoing kidney or pancreas transplant biopsy for any reason had DSA testing as a part of the biopsy visit [35, 47]. The strength of *de novo* DSA (dnDSA) was represented as the sum of the MFI of all DSA. Patients were diagnosed with dnDSA if any one of the following occurred: i) no detectable pre-transplant DSA followed by the development of new antibodies post-transplant, ii) the sum MFI increased by at least 2 fold, or iii) new alleles were detected post-transplant.

#### Infections

Post-transplant infections were categorized as bacterial or opportunistic infections (including virus, fungus, *listeria*, *nocardia*, and CMV viremia) and surgical site related. Surgical site infections were defined as any wound or intraabdominal infection within 90 days post-transplantation. Urinary tract infections (UTI) within the first-year post-transplantation were also assessed.

#### Surgical Complications

Surgical complications were categorized as either bleeding, non-bleeding or thrombotic complications (see Table 2 footnote for specific complications). Pancreatic graft thrombotic events were defined as either partial thrombosis resulting in continued graft function or complete thrombosis requiring transplant pancreatectomy or causing early graft failure within 90 days post-transplantation.

### Statistical Analysis

Differences in recipient and donor demographic factors between T1D and T2D recipients were analyzed using t-tests and Chi-square tests or Fisher’s exact tests. Multivariable Cox Proportional Hazards models, or multiple logistic regression, when appropriate, were used to investigate the association of all outcomes with diabetes types, while adjusting for recipient’s BMI, age at time of transplant, PDRI, KDPI, and induction immunosuppression. Death-censored-, rejection-free-, readmission free-, infection-free-, major surgical complication-free-, *de novo* DSA free-, thrombosis free-survival and thrombosis related to graft failure free-survival were compared between T1D and T2D using Kaplan Meier curves and log-rank tests. Post-transplant outcomes relating to the average number of episodes within the first year were analyzed using t-tests. Analyses were conducted using SAS software (version 9.4, SAS Institute Inc., Cary, NC) and p-values less than 0.05 were considered to be statistically significant.

## Results

### Study Population

A total of 345 SPKTs were categorized as 298 T1Ds and 47 T2Ds. The average post-transplant follow-up was 6.7 ± 3.6 years. Donor demographic factors were not significantly different between T1D and T2D recipients (Table 1). Several recipient demographic factors, not surprisingly, were significantly different between the cohorts. Besides the expected differences in several recipient factors such as age, BMI, ethnicity and duration of diabetes, T2D patients has lower positivity for GAD65 autoantibody and was more frequently treated with ATG and ALEM induction and early steroid withdrawal compared to T1D patients (p < .001). Lastly, there was no significant difference in the presence of pre-transplant DSA, or degree of pre-transplant DSA between the two groups.

**TABLE 1:** SPKT donor and recipient demographics.

	T1D (n = 298)	T2D (n = 47)	P-value
Donor – pre-transplant			
Age, years (mean ± sd)	29.1 ± 12.6	27 ± 12	0.28
Males	176 (59.1%)	27 (57%)	0.83
BMI, kg/m^2^ (mean ± sd)	24.0 ± 4.4	23.7 ± 4.2	0.61
Type of transplant (%DBD)	81.9%	72.3%	0.12
PDRI (mean ± sd)	1.31 ± 0.5	1.3 ± 0.4	0.59
KDPI (mean ± sd)	22.7% ± 18.7%	23.1% ± 15.2%	0.91
Pancreas cold ischemic time, hours (mean ± sd)	12.6 ± 4.1	12.8 ± 3.8	0.82
Kidney cold ischemic time, hours (mean ± sd)	13.9 ± 4.3	14.8 ± 3.8	0.23
CMV (% positive)	49%	55%	0.74
EBV (% positive)	88.4%	80%	0.27
Donor HLA Mismatch			0.67
0	2 (0.7%)	0 (0%)	
1	3 (1%)	0 (0%)	
2	12 (4%)	3 (6.4%)	
3	45 (15.1%)	4 (8.5%)	
4	86 (28.9%)	13 (27.7%)	
5	95 (31.9%)	20 (42.6%)	
6	55 (18.5%)	7 (15%)	
Recipient – pre-transplant			
Males (%)	179 (60.1%)	40(85.1%)	<.001
Recipient Race			<.001
American Indian or Alaska Native (%)	2 (0.7%)	2 (4.3%)	
Asian (%)	3 (1.0%)	4 (8.5%)	
Black or African American (%)	23 (7.7%)	10 (21%)	
White (%)	270 (90.6%)	31 (66%)	
Age at the time of diabetes mellitus diagnosis, years (mean ± sd)	13.7 ± 7.6	28.3 ± 9.1	<.001
25%–75% quartile range	8.0–18.0	21.0–35.0	
Median	12.0	27.0	
Age at the time of transplant, years (mean ± sd)	42.5 ± 9.1	47.9 ± 9.1	<.001
25%–75% quartile range	35.3–49.4	39.5–55.4	
Median	42.3	51.8	
Recipient Onset of Diabetes Greater than 30 Years			<.001
No (%)	290 (97.3%)	25 (53.2%)	
Yes (%)	8 (2.7%)	22 (46.8%)	
BMI, kg/m^2^ (mean ± sd)	25.6 ± 3.7	27.3 ± 3.4	0.004
25%–75% quartile range	23.0–27.8	24.9–29.6	
Median	25.2	27.4	
C-peptide, ng/mL (mean ± sd)	0.19 ± 0.39	3.67 ± 3.24	<.001
25%–75% quartile range	0.10–0.10	1.33–4.90	
Median	0.10	3.20	
HbA1c, % (mean ± sd)	8.38 ± 1.62	7.71 ± 1.46	0.01
25%–75% quartile range	7.20–9.30	6.65–8.80	
Median	8.30	7.70	
Family history of diabetes (% yes)	55%	85.1%	<.001
Insulin requirements pre-transplant, unit/day (mean ± sd)	39.1 ± 16.1	44.5 ± 28.4	0.21
25%–75% quartile range	27.0–50.0	20.0–60.0	
Median	37.0	40.5	
CMV (% positive)	39.4%	55.3%	0.04
EBV (% positive)	94.5%	97.9%	0.03
PRA (% mean ± sd)	7 ± 20.0	6.3 ± 16.3	0.83
Pre-transplant DSA			0.12
Negative (%)	283 (95.3%)	42 (89.4%)	
<1000 MFI (%)	8 (2.69%)	4 (8.5%)	
>1000 MFI (%)	6 (2.02%)	1 (2.13%)	
NA (%)	1 (0.003%)	0 (0%)	
Auto antibody status			
Number of tested patients	42	28	
Any auto-antibody (% positive)	73.8%	28.6%	<.001
GAD65 (% positive)	55.9%	11.1%	<.001
Insulin Ab (% positive)	63.2%	17.9%	<.001
Islet IgG (% positive)	0.0%	8%	0.14
Steroid immunosuppression			0.01
Early steroid withdrawal (%)	6 (2%)	6 (12.8%)	
Induction and maintenance (%)	266 (89.26%)	41 (87.23%)	
Induction immunosuppression			<.001
Anti-thymocyte globulin (ATG) (%)	46 (15.4%)	22 (46.8%)	
Alemtuzumab (ALEM) (%)	79 (26.5%)	10 (21.3%)	
Basiliximab (BAS) (%)	173 (58%)	15 (31.9%)	

### Patient and Graft Survival

**FIGURE 2:**
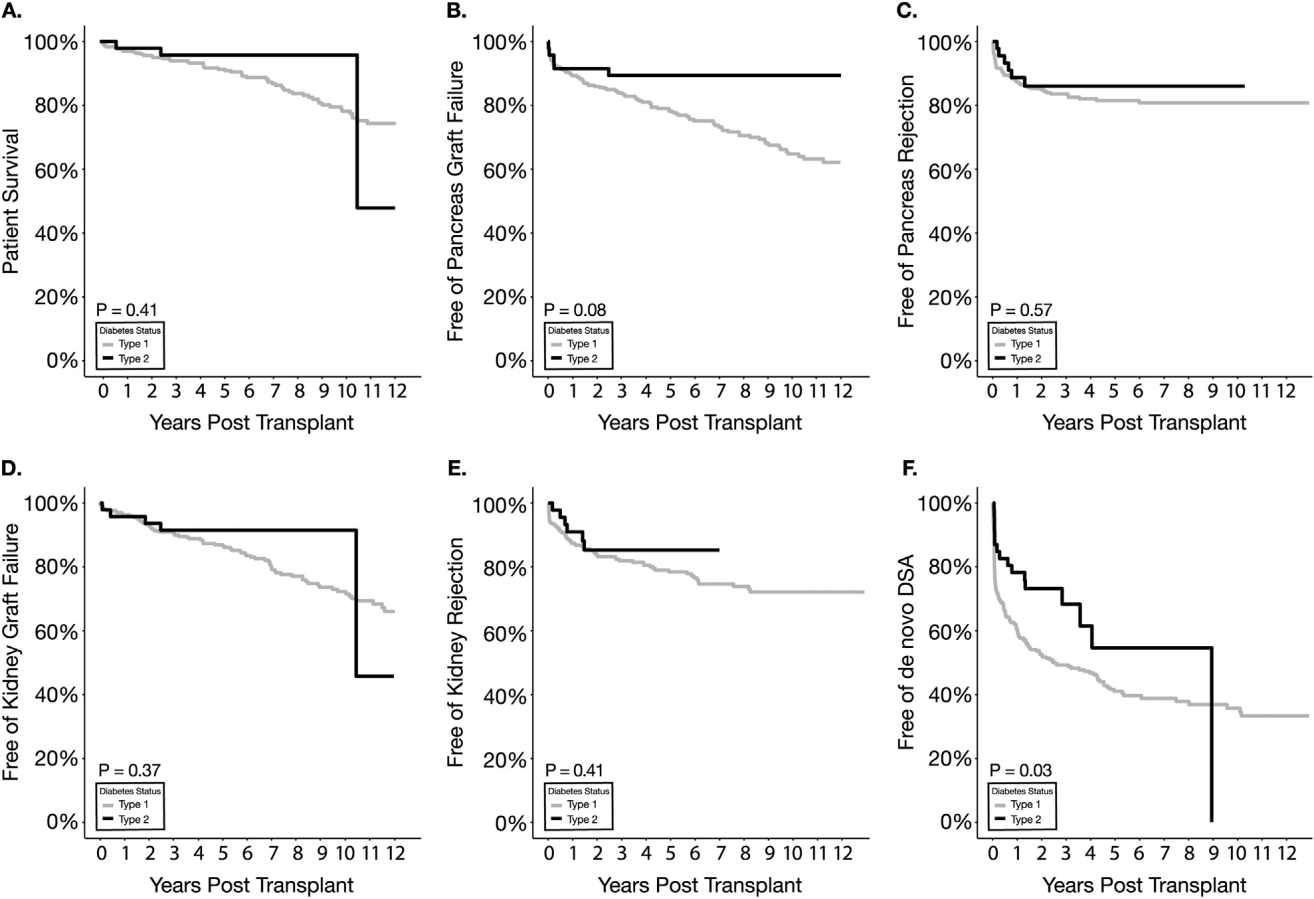
Kaplan Meier survival estimates for patient survival **(A)**, pancreas graft failure **(B)**, pancreas graft rejection **(C)**, kidney graft failure **(D)**, kidney graft rejection **(E)**, and de novo DSA **(F)**.

Patient survival (97.9% in T2D vs. 96.9% in T1D at 1 year) and pancreas graft survival (91.5% in T2D vs. 89.3% in T1D at 1 year) were not statistically significantly different between T1D and T2D SPKT recipients (Figures 2A, B; Table 2). Kidney graft survival was also not different between the two types of diabetes recipients (95.7% in T2D vs. 96.3% in T1D at 1 year) (Figure 2D; Table 2).

**TABLE 2:** Summary of major Rejection, Infection and Surgical Complication Endpoints. Kaplan-Meier Survival Estimates.

Outcomes	p-value (overall event-free survival)	1 year free of outcome (%)	
		T1D	T2D
Survival			
Patient survival	0.41	96.9%	97.9%
Pancreas graft survival	0.08	89.3%	91.5%
Kidney graft survival	0.37	96.3%	95.7%
Rejection			
Pancreas Rejection	0.57	87.3%	89.0%
Kidney Rejection	0.41	87.6%	91.2%
Post-transplant complications			
*De novo* DSA	0.03	60.6%	78.5%
Readmission	0.07	39.2%	52.7%
Infection^a^	0.12	27.6%	33.3%
Infection (UTI)	0.27	63.9%	75.8%
Major surgical complication^b^	0.84	84.5%	84.6%
Thrombosis^c^	0.46	90.7%	93.5%

^a^ Infection, unless otherwise specified, includes both bacterial and opportunistic infections.

^b^ Major surgical complication includes both bleeding and non-bleeding complication but exclude thrombosis events. Bleeding complication is defined as any of the following: intraperitoneal (intra-abdominal) bleeding, bleeding from Jackson Pratt drain site, gastrointestinal or enteric anastomotic bleeding, pancreas arterial or venous anastomotic bleeding, renal arterial or venous anastomotic bleeding, and intravesicular hematoma. Non-bleeding complications include: chylous ascites, duodenojejunostomy leak, pancreatic enzyme leak without enteric leak (capsular or retrograde via common bile duct or pancreatic duct), pancreatic pseudocyst, ureteroneocystostomy leak, ureteral stricture, and lymphocele.

^c^ Included both partial and complete thrombosis events. Specific diagnoses included partial thrombosis of the pancreatic allograft arterial or venous systems (e.g., portions of iliac Y graft, superior mesenteric artery or vein, splenic artery or vein), or complete occlusive thrombus of the pancreatic arterial or venous systems leading to pancreatectomy and early graft loss.

### Pancreas Rejection

Pancreas biopsy-proven rejection (BPR)-free survival and 1-year BPR-free survival were similar between the two types of diabetic recipients (89.0% for T2D and 87.3% for T1D) (Figure 2C; Table 2). Further stratification of rejection endpoints by grade of rejection, C4d positivity, and assessing average episodes per patient (Table 3) also failed to elucidate statistically significantly different rejection outcomes in the T1D vs. T2D recipients. Multivariable analysis (Table 4) showed that diabetes type has little association with overall pancreas BPR or other rejection subcategories. Interestingly, increasing BMI was a significant protective factor against pancreas BPR with and without Indeterminate/borderline pathology included, Grade 1 BPR, and C4d > 5% staining on biopsy (HR = 0.90, 0.89, 0.86, 0.88 respectively, all p < 0.05). Increasing PDRI was not significantly associated with any pancreas rejection endpoints. Meanwhile, increasing KDPI was significantly associated with a higher risk of pancreas BPR with Indeterminate/borderline pathology included (HR = 1.02, p = 0.03) but was not significant when excluding Indeterminate/borderline pathology. Increasing age at transplant was protective against C4d > 5% staining on biopsy (HR = 0.95, p = 0.01). Compared to BAS, both ALEM and ATG showed a trend, though not significant, to being protective toward overall pancreas BPR and BPR subcategories. Univariate analysis by induction type failed to demonstrate significant differences in index outcomes (Table 5).

**TABLE 3:** Univariate analysis for post-transplant outcomes.

	T1D (n = 298)	T2D (n = 47)	P-value
Pancreas graft rejection (Biopsy proven)			
Number patients with at least 1 rejection episode within 1st year			
BPR without Indeterminate/borderline	36 (12.6%)	5 (11%)	0.71
BPR with Indeterminate/borderline	39 (13.7%)	5 (11%)	0.57
Grade 1	22 (7.77%)	5 (11%)	0.50
Grade 2	11 (3.89%)	0 (0%)	0.18
Grade 3	6 (2.12%)	0 (0%)	0.33
Indeterminate/borderline	7 (2.48%)	0 (0%)	0.29
C4d > 5% on biopsy	20 (7.07%)	1 (2.2%)	0.22
Average episodes per patient within 1st year			
BPR without Indeterminate/borderline (mean ± sd)	0.13 ± 0.40	0.09 ± 0.29	0.41
BPR with Indeterminate/borderline (mean ± sd)	0.16 ± 0.46	0.09 ± 0.29	0.22
Grade 1 (mean ± sd)	0.08 ± 0.31	0.09 ± 0.29	0.84
Grade 2 (mean ± sd)	0.03 ± 0.18	0 ± 0	0.002
Grade 3 (mean ± sd)	0.02 ± 0.17	0 ± 0	0.03
Indeterminate/borderline (mean ± sd)	0.02 ± 0.18	0 ± 0	0.02
C4d > 5% on biopsy (mean ± sd)	0.07 ± 0.34	0 ± 0	<0.001
Kidney graft rejection (Biopsy proven)			
Number of patients with at least 1 rejection episode at 1 year	36 (12.4%)	4 (8.8%)	0.47
Average episodes per patient within 1st year (mean ± sd)	0.10 ± 0.30	0.07 ± 0.26	0.51
*De Novo* DSA within 1st year(%)	116 (39.4%)	10 (21.3%)	0.02
Readmission			
Number of patients with at least 1 readmission episode at 1 year	179 (60.7%)	22 (47%)	0.11
Average episodes per patient within 1st year (mean ± sd)	1.11 ± 1.29	0.88 ± 1.18	0.28
Number of patients at 90 days			
Any readmission	137 (46.8%)	19 (41%)	0.42
Wound-related	7 (2.43%)	2 (4.4%)	0.45
Infection-related	56 (19.2%)	10 (22%)	0.70
Rejection-related	22 (7.67%)	1 (2.2%)	0.17
Other-related	93 (31.8%)	15 (32%)	0.89
Infection (number and % of patient who have at least 1 episode)			
Bacterial infection within the 1st year	139 (47.1%)	21 (45%)	0.64
Opportunistic infection within the 1st year	141 (47.9%)	20 (43%)	0.73
Surgical site infection (within 90 days)	47 (16.0%)	6 (13%)	0.55
Non surgical site infection (within 90 days)	143 (48.2%)	18 (39%)	0.27
UTI within the 1st year	88 (30.3%)	11 (24%)	0.36
Major surgical complication (number and % of patient who have at least 1 episode)			
Any complication within 1st year	45 (15.5%)	7 (15%)	0.96
Non-bleeding fluid collection within 1st year	41 (14.2%)	7 (15%)	0.84
Bleeding complications within 1st year	7 (2.46%)	0 (0%)	0.29
Pancreas graft thrombosis event (number and % of patient who have at least 1 episode)			
Thrombosis (partial and complete) within 90 days	25 (8.52%)	3 (6.5%)	0.62
Graft failures due to thrombosis within 90 days	9 (3.09%)	1 (2.2%)	0.73

**TABLE 4:** Multivariable analysis for post-transplant outcomes.

Outcomes	Type II vs. type I		BMI		PDRI		KDPI		Age at transplant		ALEM vs. BAS		ATG vs. BAS	
	HR (95% CI) or OR (95% CI)	P-value	HR (95% CI) or OR (95% CI)	P-value	HR (95% CI) or OR (95% CI)	P-value	HR (95% CI) or OR (95% CI)	P-value	HR (95% CI) or OR (95% CI)	P-value	HR (95% CI) or OR (95% CI)	P-value	HR (95% CI) or OR (95% CI)	P-value
Biopsy proven rejection (BPR) of pancreas graft without Indeterminate/borderline	1.04 (0.42–2.55)	0.93	0.90 (0.83–0.98)	0.01	0.65 (0.25–1.66)	0.37	1.02 (0.99–1.04)	0.08	0.98 (0.95–1.01)	0.19	0.48 (0.22–1.03)	0.06	0.81 (0.38–1.72)	0.81
BPR with Indeterminate/borderline	0.88 (0.36–2.14)	0.77	0.89 (0.83–0.96)	0.003	0.57 (0.24–1.39)	0.22	1.02 (1.00–1.05)	0.03	0.98 (0.96–1.01)	0.30	0.55 (0.27–1.11)	0.09	0.89 (0.44–1.83)	0.76
Grade 1 BPR	1.40 (0.51–3.85)	0.52	0.86 (0.78–0.95)	0.003	0.89 (0.28–2.78)	0.84	1.01 (0.98–1.04)	0.48	0.98 (0.94–1.01)	0.21	0.69 (0.28–1.71)	0.42	1.28 (0.55–2.96)	0.56
C4d > 5% on biopsy	0.46 (0.06–3.52)	0.45	0.88 (0.79–0.99)	0.03	0.74 (0.21–2.65)	0.65	1.01 (0.98–1.04)	0.46	0.95 (0.91–0.99)	0.01	0.69 (0.28–1.74)	0.44	0.40 (0.09–1.71)	0.21
Kidney graft rejection (Biopsy proven)	0.96 (0.40–2.30)	0.93	1.02 (0.95–1.10)	0.54	1.11 (0.54–2.24)	0.78	1.02 (0.99–1.04)	0.07	0.97 (0.94–1.00)	0.05	0.72 (0.41–1.27)	0.26	0.40 (0.16–0.97)	0.04
*De Novo* DSA	0.70 (0.41–1.21)	0.20	0.95 (0.91–0.99)	0.02	1.38 (0.86–2.23)	0.18	0.99 (0.98–1.01)	0.80	0.99 (0.98–1.01)	0.59	0.38 (0.26–0.57)	<.001	0.63 (0.40–1.00)	0.05
Readmission	0.77 (0.50–1.20)	0.25	0.97 (0.93–1.00)	0.08	1.00 (0.68–1.47)	0.99	1.01 (0.99–1.02)	0.08	0.99 (0.98–1.01)	0.20	1.02 (0.76–1.39)	0.87	1.06 (0.74–1.53)	0.74
Infection (Any)	0.77 (0.52–1.13)	0.18	0.97 (0.94–1.00)	0.08	0.97 (0.67–1.40)	0.86	1.01 (0.99–1.02)	0.11	0.99 (0.98–1.01)	0.58	1.21 (0.92–1.61)	0.17	1.28 (0.93–1.77)	0.13
Surgical site infection^a^	0.74 (0.30–1.84)	0.52	1.02 (0.94–1.10)	0.62	1.14 (0.52–2.52)	0.73	1.01 (0.98–1.03)	0.59	0.98 (0.95–1.02)	0.31	1.44 (0.76–2.77)	0.27	1.54 (0.74–3.23)	0.24
Non-surgical site infection^a^	0.87 (0.51–1.46)	0.60	0.96 (0.92–1.01)	0.09	1.03 (0.63–1.67)	0.91	1.01 (0.99–71.02)	0.28	0.99 (0.97–1.01)	0.32	1.49 (1.03–2.14)	0.03	1.08 (0.69–1.69)	0.73
UTI	0.91 (0.51–1.62)	0.74	0.95 (0.91–0.99)	0.04	0.98 (0.57–1.67)	0.94	1.01 (0.99–1.03)	0.10	0.97 (0.95–0.99)	0.05	0.76 (0.49–1.16)	0.20	1.06 (0.66–1.71)	0.79
Major surgical complication	0.89 (0.38–2.10)	0.79	1.01 (0.94–1.08)	0.80	1.93 (0.99–3.77)	0.05	0.99 (0.97–1.02)	0.85	1.00 (0.97–1.03)	0.93	0.83 (0.43–1.60)	0.57	1.09 (0.54–2.22)	0.80
Thrombosis	0.92 (0.26–3.22)	0.90	0.88 (0.79–0.98)	0.02	2.13 (0.86–5.28)	0.10	1.00 (0.97–1.03)	0.96	1.02 (0.98–1.06)	0.41	1.22 (0.54–2.76)	0.64	0.47 (0.13–1.62)	0.23

^a^ Logistic Regression was used instead of Cox Hazard Model.

**TABLE 5:** Univariate analysis for post-transplant outcomes.

	T1D (fail/Total) (%)	T2D (fail/Total) (%)	P-value
Induction			
Anti-Thymoglobulin			
Outcomes within 1 year post-transplant			
Pancreas - BPR without Indeterminate/borderline	2/46 (4.4%)	3/22 (14%)	0.20
Pancreas - BPR with Indeterminate/borderline	3/46 (7.5%)	3/22 (14%)	0.38
Death-censored pancreas graft failure	4/46 (8.7%)	1/22 (4.5%)	0.55
Kidney rejection	1/46 (2.2%)	1/22 (4.6%)	0.58
Death-censored kidney graft failure	1/46 (2.2%)	1/22 (4.6%)	0.58
Basiliximab			
Outcomes within 1 year post-transplant			
Pancreas - BPR without Indeterminate/borderline	29/173 (16.7%)	0/15 (0.0%)	0.10
Pancreas - BPR with Indeterminate/borderline	31/173 (17.9%)	0/15 (0.0%)	0.09
Death-censored pancreas graft failure	13/173 (7.51%)	1/15 (6.7%)	0.92
Kidney rejection	26/173 (15.1%)	1/15 (6.7%)	0.39
Death-censored kidney graft failure	5/173 (2.89%)	0/15 (0.0%)	0.51
Alemtuzumab			
Outcomes within 1 year post-transplant			
Pancreas - BPR without Indeterminate/borderline	5/79 (6.4%)	2/10 (20%)	0.14
Pancreas - BPR with Indeterminate/borderline	5/79 (6.4%)	2/10 (20%)	0.14
Death-censored pancreas graft failure	10/79 (13%)	2/10 (20%)	0.55
Kidney rejection	9/79 (11%)	2/10 (20%)	0.40
Death-censored kidney graft failure	0/79 (0%)	1/10 (10%)	0.005

### Kidney Rejection

Overall kidney rejection-free survival between T2D and T1D was not significantly different (p = 0.41) (Figure 2E; Table 2). In univariate analysis, the rate of kidney BPR within the first year was 8.8% in T2D recipients vs. 12.4% in T1D recipients (p = 0.47) (Table 3). The lack of association between diabetes type and kidney graft rejection was confirmed in multivariable analysis (Table 4). Unlike for pancreas graft rejection, neither BMI nor KDPI were significantly associated with an increased risk of kidney rejection. Older age at transplant was marginally protective against rejection (HR = 0.97, p = 0.05) (Table 4). Compared to BAS, ATG was significantly associated with decreased kidney BPR (HR = 0.40, p = 0.04).

### De Novo DSA

Overall dnDSA-free survival between T2D and T1D was significantly different (p = 0.03) (Figure 2F; Table 2). A significantly lower incidence of dnDSA was observed in T2D (21%) compared to T1D (39%) within the first year (p = 0.02) (Table 3). Multivariable analysis, however, showed that type of diabetes has no association with developing *de novo* DSA while suggesting that increasing BMI was protective against such an outcome (HR = 0.95, p = 0.02) (Table 4). Regarding peri-operative induction agent use, compared to BAS, ALEM was significantly associated with decreased development of dnDSA (HR = 0.38, p < .001).

### Readmission

**FIGURE 3:**
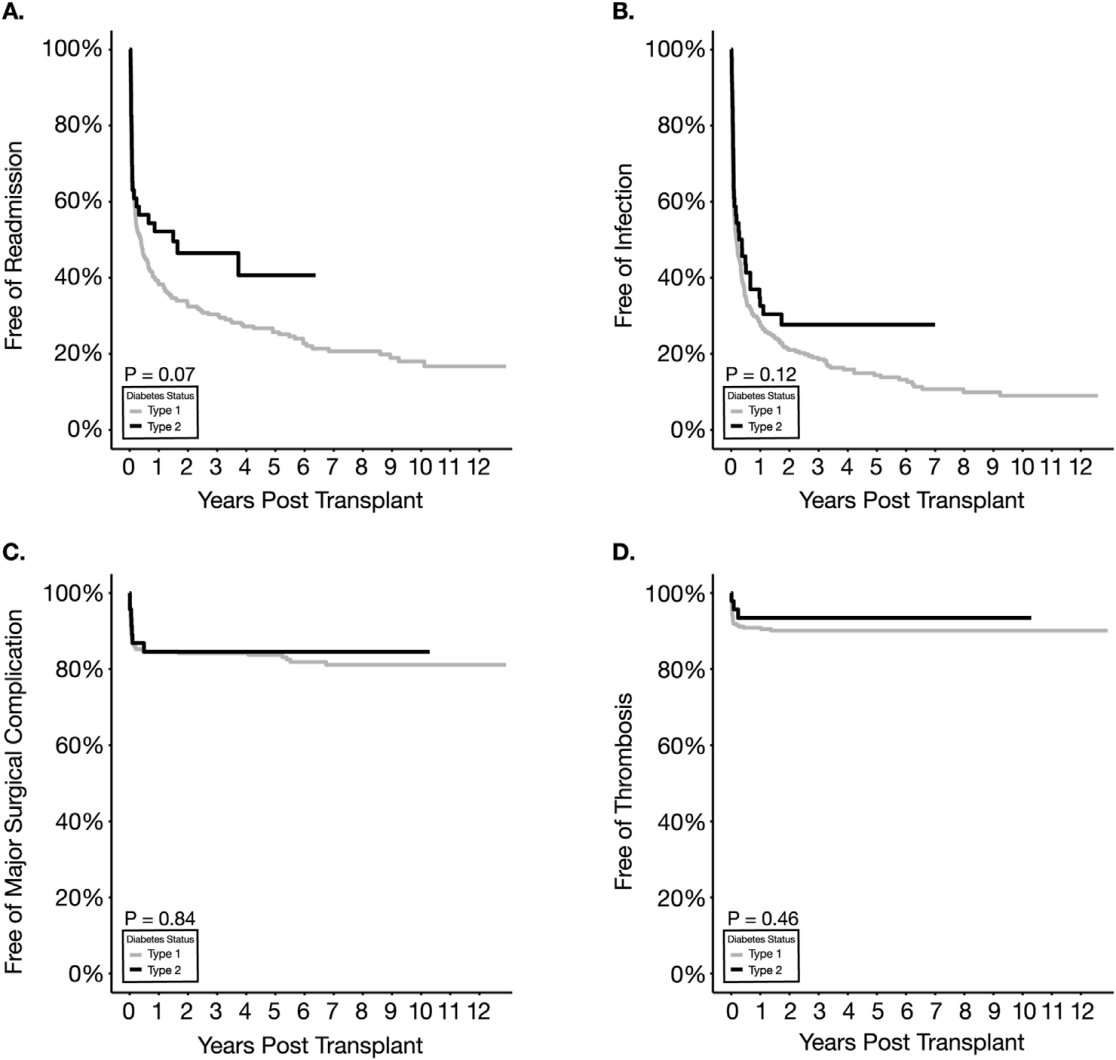
Kaplan Meier survival estimates for readmission **(A)**, infection **(B)**, major surgical complications **(C)** and thrombosis **(D)**.

Kaplan-Meier analysis of freedom from readmission showed no difference between the two types of diabetes (p = 0.07) (Figure 3A; Table 2). The percentage of readmissions within the first-year post-transplantation was not significantly different when comparing T2D with T1D recipients (47% vs. 60.7%, p = 0.11) though there was a trend to fewer readmissions in T2D recipients (Table 3). Positive trends in favor of T2D were also identified in the average number of readmission episodes per patient within the first year as well as overall readmissions within the first 90 days, though the results did not reach statistical significance. In the multivariable analysis (Table 4), neither type of diabetes nor other factors were associated with overall readmission risk.

### Post-Transplant Infections

No statistical difference was observed between T2D and T1D recipients with respect to overall infection-free survival (p = 0.12) and UTI-free survival (p = 0.27) (Figure 3B; Table 2). There was no significant difference between the two types of diabetic recipients regarding the sub-categories of infection (Table 3). Multivariable analysis also supported the similarity between the two types in overall infection, UTI, surgical- and non-surgical site infection (Table 4). Increasing BMI was significantly associated with decreased risk of UTI (HR = 0.95, p = 0.04), whereas using ALEM was significantly associated with an increased risk of non-surgical site infection (HR = 1.49, p = 0.03).

### Major Surgical Complications

Overall surgical complication-free survival was not significantly different between the two groups (Figure 3C; Table 2). A significant difference was not observed in the frequency or distribution of major surgical complications or subtypes (i.e., bleeding and non-bleeding) within the first -year post-transplantation between T1D and T2D recipients (Table 3). Multivariable analysis also showed that none of the variables tested, including diabetes types, were significantly associated with an increased risk of major surgical complication (Table 4).

### Thrombosis Events

No difference in thrombosis-free survival was detected between T1D and T2D recipients with 1-year survivals of 90.3% and 94.8% in T1D and T2D respectively (Figure 3D; Table 2). Within the first 90 days post-SPKT, partial pancreatic thrombotic events and pancreas graft failures secondary to thrombosis were also not different between T1D and T2D in both univariate and multivariable analyses (Table 3, 4). Interestingly, on multivariable analysis, increasing BMI was significantly associated with a lower risk of thrombosis (HR = 0.88, p = 0.02).

## Discussion

Whereas the majority of studies focus on patient and graft survival outcomes between T1D and T2D recipients, few address key infectious, surgical, and immunological outcomes. The current study addresses this gap and demonstrates that similar post-transplant outcomes, such as the incidence of acute BPR, readmissions, infections, UTIs, thrombosis, and other major surgical complications can be achieved between T1D and T2D SPKT recipients. Also, consistent with findings from previous studies demonstrating improvement in patient survival with advancing eras [9, 10, 43], the present study demonstrates acceptable and comparable patient-, pancreas allograft- and kidney allograft-survival in T2D versus T1D SPKT recipients.

Organ transplant recipients whose primary etiology of organ failure is autoimmune in nature may have higher rates of rejection and recurrence, especially in kidney transplantation [19–23] and liver transplantation [24–30]. However, a UNOS registry review did not find a significant association of rejection between T2D and T1D when combining kidney and pancreas rejection outcomes [10]. This study has the caveat however that kidney and pancreas rejection were not analyzed separately, and the majority of centers did not perform routine pancreas allograft biopsies in SPKT recipients, thereby potentially leading to underreporting of pancreas rejection. Thus, we posited that T1D SPKT recipients may experience higher rates of pancreas rejection than T2D SPKT recipients given the autoimmune nature of diabetes in the former. However, we did not observe a significantly different 1-year pancreas, or kidney, BPR rate in T2D vs. T1D patients. The overall rates of rejection in our population are consistent with those previously reported in the literature (4%–38%) [48–53]. The current study provides greater granularity particular to rejection type and severity compared to prior studies [6, 9, 10, 14, 15, 52]. These prior studies, additionally, did not meticulously categorize T1D and T2D recipients [14, 15, 54], assess pancreas BPR separately from kidney BPR [10, 14, 15, 55, 56] or specifically look at pancreas BPR [6, 9, 52, 57]. Thus, the current study adds a more comprehensive assessment of the rejection risk confronting T2D SPKT recipients. It also suggests a very low overall incidence of pancreas antibody-mediated rejection (ABMR) based on the ∼6% overall incidence of C4d>5% staining on biopsies in both T1D and T2D recipients, which is consistent with previously reported data [50].

While not definitive, our data does suggest a possible signal with regard to more rejection in T1D recipients. For example, we observed a greater number of episodes of Grade 2-3 ACR, indeterminate ACR, and C4d+ rejection, and numerically more patients with these rejection diagnoses within the first year in T1D patients. Moreover, we observed a higher incidence of dnDSA in T1D patients compared to T2D patients. Thus, this type of diabetes may be associated with an increased risk of pancreas rejection endpoints. Though we observed a higher rate of pancreas rejection signals by univariate analysis, we failed to detect a significant difference in multivariable analyses. Given the lack of major differences between T1D and T2D SPKT recipients, the current study suggests that a primary autoimmune pathology does not pose a substantially increased risk of BPR, nor does it suggest T2D confers higher rates of pancreas or kidney BPR. Thus, the type of diabetes thus should not affect candidacy for SPKT from the rejection perspective.

Similar patient and graft survival outcomes have been described with both T-cell depleting and non-depleting agents for SPKT [53, 58–60]. Overall, lower rates of early acute rejection have been described in SPKT with T-cell-depleting agents versus non-depleting agents [53]. Comparing types of T-cell-depleting therapies, ALEM versus ATG has been associated with comparable surgical complications, readmissions, thromboses, and bleeding [61]. These studies however involve very few T2D recipients. The results of our study are congruent with these findings and indicate that, compared to BAS, ALEM induction might be beneficial for pancreas graft rejection and was associated with lower risk of dnDSA development, while ATG induction was associated with reduced kidney graft rejection. We also did not find an association between ALEM and kidney graft rejection, consistent with Sampaio et al [10]. Induction trends in our cohort are also consistent with those reported in T2D recipients represented in registry data, with an increasing trend toward use of T-cell-depleting antibodies in more recent eras [9]. Larger cohorts of T2D SPKT recipients are needed to make definitive conclusions regarding any differences in the rejection rate between induction regimens based on diabetes type.

The development of dnDSA after pancreas and SPK transplantation has been identified as a significant risk factor for pancreas and kidney rejection, and for graft failure [33–35]. We demonstrated a significantly lower incidence of dnDSA within the first year in T2D versus T1D SPKT recipients. This result may be explained by differences in induction immunosuppression mentioned earlier (i.e., more BAS induction in T1D vs. T2D recipients), and therefore should not necessarily be construed as definitively indicating T2D SPKT recipients would require less intensive immunosuppression or less vigorous postoperative-immune monitoring, though these benefits remain a possibility.

Previous analysis of SPKT registry data from over a decade ago [10] and more recent UK registry data [55] has suggested that the type of diabetes did not significantly impact the rate of surgical complications including abscess formation, anastomotic leak, pancreatitis, and primary non-function. Obesity, frequently associated with T2D, on the other hand, has been associated with increased risk of postoperative infections, a need for postoperative invasive procedures [62, 63], increased risk of patient death, pancreas graft loss, and kidney graft loss [39]. Our findings demonstrate no difference in risks of major surgical complications (bleeding and non-bleeding), surgical site infections, incidental image-identified pancreatic graft thrombotic lesions, and pancreatic graft losses secondary to thrombosis in T2D vs. T1D recipients. In the absence of significantly worse infectious and surgical complications and similar rejection rates between T2D and T1D SPKT recipients, it seems very reasonable to continue to offer selected IDDM/CKD patients an SPKT regardless of their diabetes labels. Prospective trials would also be valuable to definitively compare efficacy and safety outcome endpoints, but await a significant multi-center effort to accrue a sufficient number of patients. In the meantime, we recommend a careful and systematic center-specific approach to offering SPKT to T2D/CKD patients.

Given the rising rates of T2D-associated CKD and obesity, safe criteria for SPKT in the T2D/CKD population should be established [64, 65]. Though we found some marginal protective effect associated with older age with regard to pancreas and kidney rejection, elderly patients tend to preform poorly due to having more comorbidities. UNOS/OPTN policy still requires patients to be insulin-dependent, though weight or BMI restrictions were recently eliminated [44]. Consequently, the indication for SPKT for T2D and CKD at most centers in the US is quite narrow and the majority of T2D/CKD patients presenting to centers are not considered candidates for SPKT but are generally offered a kidney transplant alone. Morbidly obese patients with CKD who do not require insulin most likely have residual beta cell mass, and their diabetes could be reversed by bariatric surgery [66–70]. However, if they have undetectable or minimal C-peptide, their diabetes is unlikely reversed by bariatric surgery alone. CKD patients whose diabetes is controlled by non-insulin oral or injectable agents, diet or exercise are not eligible for pancreas transplantation currently in the US based on allocation policy. However, it is well understood by the transplant community that once they receive a kidney transplant and the requisite immunosuppression, the patient’s diabetes will worsen and ultimately require long-term insulin for control. In this situation, they may benefit from a pancreas-after-kidney transplant, but would it be reasonable to offer a “preemptive” SPKT to this population, preempting their requirement for insulin, just as we offer kidneys preemptively in patients with CKD prior to dialysis? Understanding the relative mortality risk of T2D/CKD waiting list patients who are controlled without insulin to those who are on insulin may support future policy decisions.

We recognize potential limitations to the broader applicability of the results presented here given the non-randomized, single-center, and retrospective nature of our study. Despite using an objective multiparametric approach to classify diabetes type, mis-categorization is possible as not all patients fit neatly into the classically defined T1D and T2D categories, though we believe that this approach is more holistic and objective. We also acknowledge that dnDSA and rejection may still develop after our 1 year minimum follow-up period. Therefore, to ensure valid conclusions can be made, we limited our incidence analysis of immunological, surgical, and infectious complications to the first year or less so that every patient had an equal chance to realize these complications. Consequently, we cannot describe medium or longer-term outcomes relative to these complications. Lastly, our T2D population is relatively small compared to registry data, albeit one of the larger single-center experiences presented to date. However, we feel that the granularity of our data, the recent cohort, and the greater homogeneity of candidate selection, surgical technique, immunosuppression, and post-operative practices at a single center than exists in registry data are benefits to teasing out differences between these populations and to provide updated information. Nonetheless, we believe these data provide useful guidance by comprehensively examining immunological, infectious, and surgical complications after SPKT in T2D recipients.

In conclusion, with the increasing prevalence of T2D related ESRD and an increasing trend of SPKT performed in T2D(9) this study found similar outcomes regarding rejection, major surgical complications, infections, and readmissions between SPKT T1D and T2D recipients. It further demonstrates the success that SPKT can achieve in carefully selected T2D recipients, and provides valuable reassurance to the transplant community for continued careful protocolized application of SPKT to low cardiovascular risk T2D/CKD patients.

## Data Availability

The raw data supporting the conclusions of this article will be made available by the authors, without undue reservation.

## Abbreviations

ALEM, Alemtuzumab; ATG, Antithymocyte globulin; BAS, Basiliximab; BMI, body mass index; BPR, biopsy proven rejection; CIT, cold ischemia time; CMV, cytomegalovirus; dnDSA, *de novo* anti-HLA donor specific antibody; DCD, donation after circulatory death; ESRD, end stage renal disease; KDPI, kidney donor profile index; OPTN/UNOS, Organ Procurement and Transplant Network/United Network for Organ Sharing; PDRI, pancreas donor risk index; SPKT, simultaneous pancreas kidney transplant; STAR, Standard Transplant Analysis and Research; T1D, Type I diabetes mellitus; T2D, Type II diabetes mellitus; UW, University of Wisconsin.
